# Alert plan-application “MySolutions” - lived experiences of users with a serious mental illness

**DOI:** 10.1192/j.eurpsy.2022.360

**Published:** 2022-09-01

**Authors:** J. Bom, J. Bruintjes, B. Sportel

**Affiliations:** 1 GGZ Drenthe, Psychosis Department, Assen, Netherlands; 2 GGZ Centraal, Psychotic Disorders, Harderwijk, Netherlands; 3 GGz Drenthe, Psychosis Department, Assen, Netherlands

**Keywords:** users experience, Serious mental illness, relapse prevention plan, alert plan

## Abstract

**Introduction:**

In daily life an alert or relaps prevention plan can be a helpful tool in preventing patients with severe mental illness (SMI) from relapse. However, patients often find it hard to keep the paper version close by. A smartphone version could be a solution. “MySolutions” is a webapplication providing the possibility to add e.g. pictures or music to the alert plan, which could be helping in time of need.

**Objectives:**

To describe the lived experiences of patients with (SMI) with the webapplication ‘MySolutions’ and get insight in the helping and hindering characteristics of the application.

**Methods:**

Qualitative research in a fenomenological framework. Eight interviews were held with outpatients with SMI. All interviews where methodically analyzed using the steps of Colaizzi (1978).

**Results:**

In general, users were enthousiastic about the look and feel of the application. Using the application was considerd easy. Lived experiences of participants displayed the following themes: Autonomy, Acceptance, Frustration, Self confidence, and Reassurance. By practicing and adding photos and music, they perceived the webapplication to be a personal aid tool for experienced problems related to mental vulnerability in daily life. Participants also reported more difficulties in using the application in times of dysregulation.

**Conclusions:**

The webapplication can be a valuable addition to the alert plan for people with SMI due to the possibility of personalization and the fact it is always available on a mobile phone. The application seems particularly suiting for people in a stabile phase. Future research should focus on phase of recovery in relation to use of the application.

**Disclosure:**

No significant relationships.

